# Evaluation of the V-gel^®^ Advanced Supraglottic Airway Device Across Different Ventilatory Modes in Anaesthetised Cats

**DOI:** 10.3390/vetsci12121112

**Published:** 2025-11-22

**Authors:** Jaime Viscasillas Monteagudo, Esther Martinez Parrón, Jose Manuel Gómez-Silvestre, Maria de los Reyes Marti-Scharfhausen, Eva Zoe Hernández Magaña, Alvaro Jesús Gutiérrez-Bautista, Ariel Cañon Pérez, Agustín Martínez Albiñana, José Ignacio Redondo

**Affiliations:** 1 AniCura Valencia Sur Hospital Veterinario, Av. de Picassent, 28, 46460 Silla, Valencia, Spain; 2 Esthervetequina. C/Noruega 5, 07840 Santa Eulalia del Rio, Ibiza, Spain; esthervetequina@outlook.es; 3 Village Vet Hampstead, 11 Belsize Terrace, Belsize Park, London NW3 4AX, UK; jmgomezs98@gmail.com; 4 AniCura Indautxu Hospital Veterinario, Bekoetxebarri Bidea, 5F, 48950 Erandio, Bizkaia, Spain; reyes.marti@anicura.es; 5 Departamento de Medicina y Cirugía Animal, Facultad de Veterinaria, Universidad Cardenal Herrera-CEU, CEU Universities, C/Tirant lo Blanch, 7, 46115 Alfara del Patriarca, Valencia, Spain; eva.hernandezmagana@uchceu.es (E.Z.H.M.); nacho@uchceu.es (J.I.R.); 6 The Royal (Dick) School of Veterinary Studies, The University of Edinburgh, Easter Bush Campus, Midlothian EH25 9RG, UK; agutier6@ed.ac.uk; 7 Experimental Surgery Unit, Institut de Recerca, Hospital Vall d’Hebron, VHIR Edifici Mediterrània, Pg. de la Vall d’Hebron, 129, Horta-Guinardó, 08035 Barcelona, Barcelona, Spain; ariel.canon@vhir.org; 8 AniCura Aitana Hospital Veterinario, C/de Xirivella, 16, 46920 Mislata, Valencia, Spain; agustin.martinez@anicura.es

**Keywords:** cat, leakage, complications, supraglottic device, ventilation, V-gel advanced

## Abstract

Endotracheal intubation in cats carries risks; supraglottic airway devices (SADs) offer a less invasive alternative. This prospective clinical study evaluated the V-gel^®^ advanced in cats undergoing ovariohysterectomy, with the primary aim of intra-anaesthetic gas leakage; secondary aims were ease of placement, complications, and the effect of device re-use. Spirometric inspired/expired tidal volumes were recorded under predefined ventilatory settings; leakage was calculated and analysed. Of 52 cats enrolled, 47 were analysed. Leakage occurred in 13% during spontaneous breathing and rose to 41.8% with Continuous Positive Airway Pressure (CPAP) 5 cmH_2_O; by contrast, controlled mechanical ventilation showed lower leakage, with the smallest values at Peak Inspiratory Pressure (PIP) of 12 cmH_2_O and 16 cmH_2_O (2.1% and 6.5%, respectively). Re-used devices leaked less than new ones (*p* = 0.003). Placement by students was straightforward after adequate depth was achieved. Complications included mild regurgitant material in three cats and pulmonary aspiration in two (one euthanised, one discharged after supportive care). The V-gel^®^ advanced enabled rapid airway management with leakage influenced by ventilation mode, airway pressure, and device re-use. Findings support cautious use during spontaneous breathing or CPAP, attention to fasting and fresh-gas flows, and further controlled comparisons with prior V-gel^®^ models and endotracheal intubation.

## Simple Summary

Cats need safe ways to keep their airway open during surgery, but placing an endotracheal tube in the trachea can sometimes cause harm. We tested a supraglottic device that sits above the larynx to see how well it prevents unwanted air leaks and how easy it is to use. In a real surgical programme for street-living cats, supervised students placed the device and we compared the air breathed in with the air breathed out under several breathing settings. Among fifty-two cats, forty-seven were fully assessed. Small leaks appeared in about one in eight cats during natural breathing and were more common when a steady pressure was used to keep the airway open; leaks were lower when a machine delivered each breath, especially at higher set pressures. Re-used devices leaked less than new ones, but any re-use demands careful cleaning and safety checks. Placement was generally straightforward. Complications were uncommon but included regurgitation and two cases of stomach contents entering the lungs. These findings can help veterinary teams choose breathing methods and handling steps that improve safety for cats during routine operations.

## 1. Introduction

Endotracheal intubation is a well-established technique for airway management in cats undergoing general anaesthesia. However, it is not without risk and has been identified as a contributor to feline anaesthetic mortality [1,2]. Reported complications include laryngospasm during intubation or following extubation, oedema [1], arytenoid tears [3], tracheal mucosal injury [4] and, in severe cases, tracheal rupture [3]. Accordingly, endotracheal intubation in cats should be performed with particular care and by appropriately trained personnel.

In human anaesthesia, less invasive alternatives to endotracheal intubation have been developed for airway management in anaesthetised patients. The supraglottic devices have been used not only for minor procedures but abdominal surgery [5,6]. These devices must provide an adequate laryngeal seal for two principal reasons: (1) to permit effective ventilation without leakage between inspired and expired volumes; and (2) to minimise the passage of secretions or fluids into the airway. Supraglottic airway devices (SADs) fulfil these requirements and have demonstrated substantial efficacy across multiple studies [7,8,9]. Although designed for humans, SADs have also been applied in several animal species, including rats, rabbits, dogs and pigs [10,11,12].

The V-gel^®^ (Docsinnovent, UK) was the first SAD purpose-designed for veterinary use and is anatomically tailored for cats, rabbits and dogs [13]. In feline studies, V-gel^®^ has shown several advantages over endotracheal intubation: easier and faster placement by inexperienced operators [14]; placement at a lighter plane of anaesthesia, allowing reduced induction drug dosing and potentially fewer adverse effects [15]; less stridor and discomfort during recovery [14]; and equal or reduced leakage during ventilation [14,16,17].

A new iteration, V-gel^®^ advanced, has recently been introduced. According to the manufacturer, key enhancements include reduced connector dead space; improved fixation features to limit displacement and rotation; lateral tabs to facilitate positioning; a re-designed laryngeal cup intended to improve seal and eliminate the need for an inflatable cuff; and an oesophageal sealing plug. The device is intended for single use in line with human healthcare hygiene standards. To date, and to the best of the authors’ knowledge, only two studies using the V-gel^®^ advanced have assessed device position on CT imaging [18,19]. However, there are no clinical studies evaluating this new design in cats assessing its performance during abdominal surgery and across different ventilatory setting.

Accordingly, the primary objective of this study was to quantify gas leakage during ventilation under general anaesthesia when using the V-gel^®^ advanced. Secondary objectives were to evaluate the ease of device placement, to describe any complications observed during abdominal surgery related to the use of this device, and to compare leakage between new and re-used devices. We hypothesised, in line with these objectives, that intra-anaesthetic gas leakage with the V-gel^®^ advanced would be comparable to that reported for the previous V-gel^®^ model; that the device would be easy to place; that few complications would occur in this surgical context; and that leakage would be lower with new devices than with re-used ones.

## 2. Materials and Methods

The study was approved by the Ethics Committee of University CEU-Cardenal Herrera (CEEA 20/017). It was a prospective clinical study conducted in an unowned free-roaming cat colony enrolled in an elective ovariohysterectomy programme at the university’s Veterinary Teaching Hospital. Final-year veterinary students performed the anaesthesia and surgery under the direct supervision of specialist lecturers.

Cats were admitted 12–24 h before surgery. Food was withheld for 8 h, with water available ad libitum. Deep sedation/anaesthesia was administered intramuscularly using dexmedetomidine (Dexdomitor^®^, Zoetis, Madrid, Spain) at 15 μg/kg, methadone (Semfortan^®^, Dechra, Barcelona, Spain) at 0.3 mg/kg, and ketamine (Ketamidor^®^, Richter Pharma, Wels, Austria) at 5 mg/kg.

The V-gel^®^ advanced (Docsinnovent, Hemel Hempstead, UK) size was selected according to the manufacturer’s body-weight guidance. Each device was colour-labelled prior to study commencement to track re-use count. Randomisation between a new or previously used supraglottic device was performed by drawing a slip of paper from an opaque envelope. Previously used supraglottic devices were cleaned and disinfected after each use. Before placement, a visual inspection was conducted to confirm the absence of any physical defects in the device such as a weakened interface between the connector and the remainder of the device, the presence of cracks or roughened areas on either the external or internal surfaces, or residual contamination/debris. After a brief standardised demonstration by the anaesthesia lecturer, students positioned cats in sternal recumbency with an assistant stabilising the head, gently exteriorised the tongue, and advanced the device. If placement was not tolerated, a top-up of one of the sedative/analgesic agents was intramuscularly administered at the lecturer’s discretion. Correct placement was confirmed by an appropriate capnography waveform and the number of attempts recorded; unsuccessful attempts were repeated. Devices were secured using gauze around the neck. At this point, a mixture of oxygen and sevoflurane was administered via a paediatric circle system at a flow and concentration selected case by case by the student and anaesthesia lecturer.

**Figure 1:**
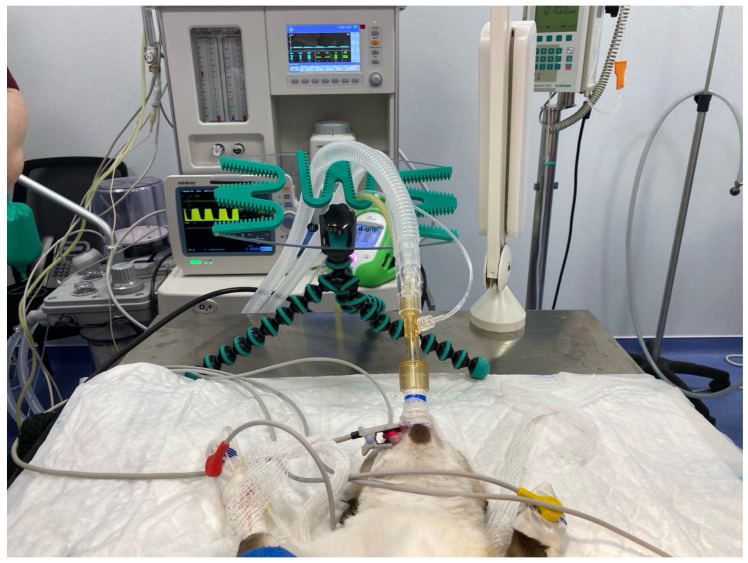
Picture showing the positioning of the cat with the spirometer and the device to hold the breathing system ready to start with the different ventilatory settings.

A cephalic venous catheter was then placed and lactated Ringer’s solution (Lactato-RingerVet^®^, B. Braun VetCare, Barcelona, Spain) was infused at 5–10 mL/kg/h. Anaesthesia was maintained with oxygen and sevoflurane. For surgery, cats were positioned in dorsal recumbency. To avoid device rotation during repositioning (as previously reported), the breathing system was briefly disconnected; after repositioning, it was reconnected and capnography re-verified. The breathing system was then supported with a D-grip^®^ tube holder (Docsinnovent, UK) (Figure 1). Monitoring included pulse oximetry, capnography, electrocardiography, and temperature using a multiparametric monitor (GE B450, GE Medical, Madrid, Spain). Non-invasive arterial blood pressure was measured by oscillometry (Suntech VET30, Morrisville, NC, USA).

**Figure 2:**
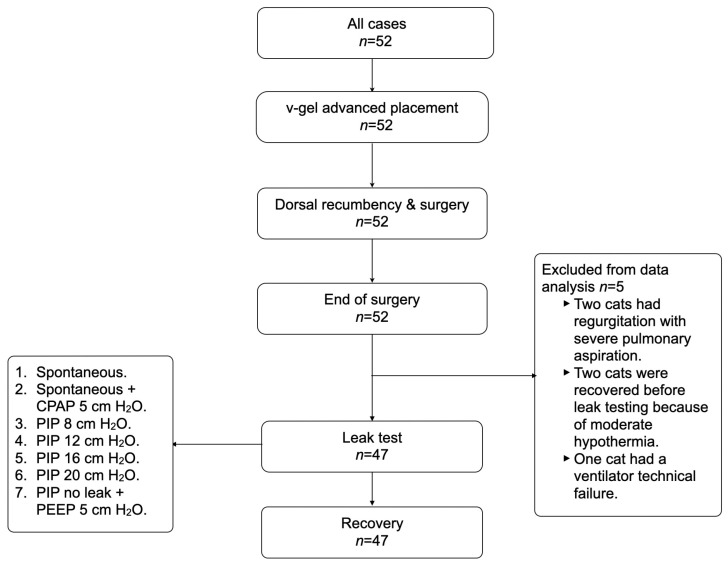
Basic sequence of the steps performed in this study and the different ventilatory modes tested in each animal.

At the end of surgery, cats remained in dorsal recumbency, and a disposable paediatric spirometry sensor (Pedi-lite+, GE Healthcare, Chalfont St Giles, UK) was fitted (Figure 1). The spirometry module was calibrated before data collection. All leak tests were performed using the same anaesthetic machine (Mindray WATO EX-30, Shenzhen, China) and the same multiparametric monitor (GE B450, GE Medical, Spain). Ventilatory settings assessed are summarised in Figure 2. For each setting, inspired (VTi) and expired (VTe) tidal volumes were recorded and the percentage leakage was calculated when a discrepancy was present. In line with previous literature, a difference ≥20% between VTi and VTe was defined as clinically significant leakage [15]. Ventilatory parameters were recorded by one operator while a second operator performed the procedural steps under study. The ventilatory settings were applied in a fixed sequence (not randomised). The first settings assessed were spontaneous ventilation and spontaneous ventilation with 5 cmH_2_O continuous positive airway pressure (CPAP); this CPAP level was achieved by adjusting the adjustable pressure-limiting (APL) valve and verified by the anaesthetic machine manometer. Thereafter, animals were switched to pressure-controlled mechanical ventilation with peak inspiratory pressure (PIP) targets of 8, 12, 16, and 20 cmH_2_O, using an I:E ratio of 1:2 and a respiratory rate of 10 breaths/min. For each animal, once the PIP at which leak occurred was identified, an additional measurement was obtained at the highest PIP without leak, this time with 5 cmH_2_O positive end-expiratory pressure (PEEP) applied. A minimum of 10 breaths preceded every measurement to ensure steady-state ventilation. For each setting, three consecutive readings were taken, and their mean was used for analysis. At the end of each day, all values were transcribed into an excel spreadsheet and stored for final analysis.

Collected variables included bodyweight (kg), V-gel^®^ advanced size, number of device re-uses, number of attempts to achieve correct placement, need for sedative/analgesic top-ups prior to placement, spirometric volumes, any anaesthesia-related complications attributable to the V-gel^®^ advanced and anaesthesia time (in minutes), defined as the period from the placement of the supraglottic device until its removal. Complications attributed to the supraglottic device included capnography alterations, pulse oximetry showing real hypoxemia (S_p_O_2_ less than 95%), regurgitation, tongue swelling, or any other problem directly affecting device performance. Potential peri-anaesthetic events such as hypotension or hypothermia were treated and documented in the anaesthetic record but were not considered consequences of device use. In addition, any complications considered unrelated to the device were documented. Ease of placement was defined by the number of attempts required to obtain an appropriate capnography waveform once a sufficiently deep plane of anaesthesia had been achieved. Placement was classified as easy if successful within one or two attempts, and difficult if more than two attempts were required. An attempt was defined as insertion of the device followed by assessment of a correct capnography waveform in situ. Following completion of leak tests, anaesthesia was discontinued and recovery monitored in-hospital for 24 h. Devices were manually cleaned by external and intraluminal brushing under running water to remove visible residues, then immersed in 7.5% hydrogen peroxide for 30 min. After immersion, they were thoroughly rinsed with clean water and allowed to air-dry. This protocol mirrors the institution’s standard procedure for endotracheal tubes. Cats were discharged 24 h post-operatively if no postoperative complications related to the surgery were observed.

Statistical analyses were conducted using R (version 4.0.4). Normality was assessed using the Shapiro–Wilk test with visual inspection of histograms. Where distributional assumptions were not met, non-parametric procedures were applied. For within-cat comparisons of inspired/expired tidal volumes across ventilatory settings (Sp, Sp + CPAP, PIP 8/12/16/20 cmH_2_O, and the highest non-leak PEEP condition), overall differences were tested using the Kruskal–Wallis test and, when indicated, pairwise Wilcoxon rank-sum tests with Holm correction for multiplicity. As a robustness check, we additionally used trimmed-means repeated-measures ANOVA with rmmcp post hoc contrasts from WRS2. Leak was defined a priori as VTi–VTe ≥ 20% (yes/no). The primary inferential analysis for leak occurrence used a mixed-effects logistic regression with a random intercept for cat to account for repeated settings. Fixed effects included ventilatory setting and device condition (new vs. re-used), with their interaction prespecified. Device size (C2 vs. C3) was included in sensitivity analyses. We report odds ratios (ORs) with 95% confidence intervals (CIs) from profile likelihood and likelihood-ratio tests for global effects. Assumptions were examined through simulation-based residual diagnostics, checks for overdispersion, variance inflation factors (indicating multicollinearity), and inspection of influential observations. A simple chi-square comparison of leak rates between new and re-used devices is provided as a descriptive complement only. Two-sided *p*-values < 0.05 were considered statistically significant. An a priori power calculation indicated that a sample of approximately 47 observations would provide 80% power at α = 0.05 to detect a medium effect size. The cohort comprised consecutive teaching-hospital cases and therefore represents a convenience sample; implications for precision and external validity are discussed.

## 3. Results

Fifty-two cats were enrolled in the study; leak-test data were obtained from 47. Two cats were withdrawn from the study owing to regurgitation with severe pulmonary aspiration, two were recovered before leak testing because of moderate hypothermia (less than 35 °C), and one was excluded due to ventilator technical failure. Of the 47 analysed cats, complete datasets across all ventilatory settings were available for 39; in the remaining eight, one or more settings could not be collected for reasons unrelated to the protocol.

Bodyweight, need for sedative/analgesic top-ups before placing the device, number of placement attempts, V-gel^®^ advanced size and number of new versus re-used devices are summarised in Table 1.

**Figure 3:**
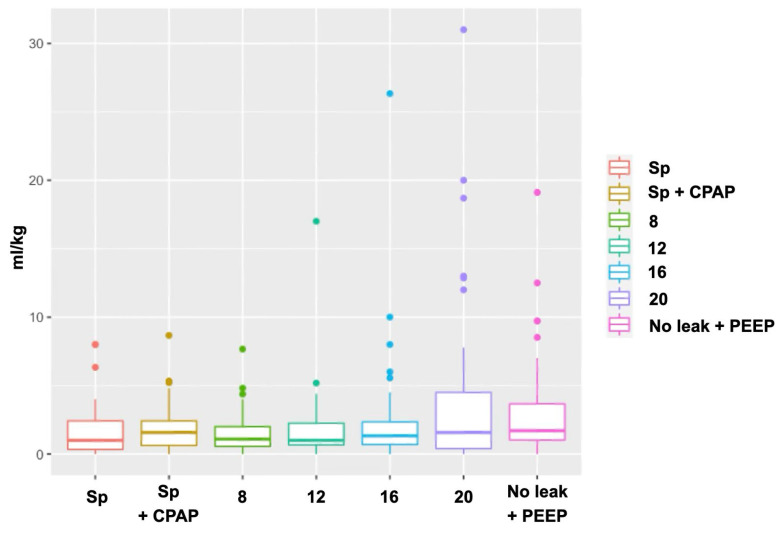
Graph depicting leakage volume (mL/kg) across ventilatory settings.

**Figure 4:**
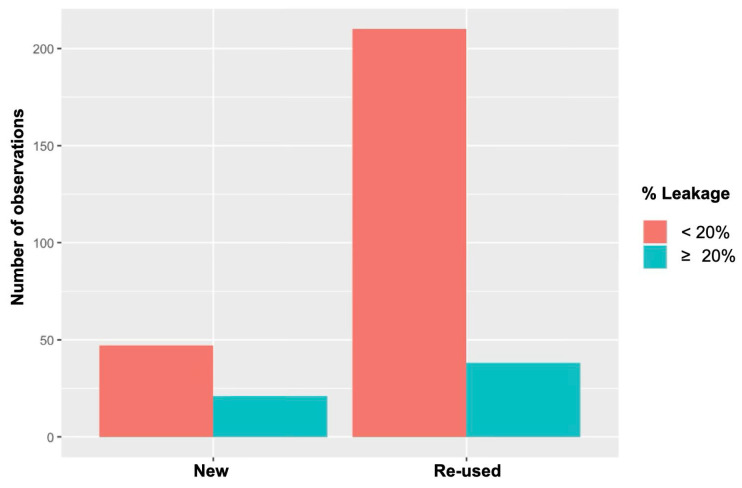
Frequency of new versus re-used supraglottic devices. For each category, bars are split into cases without leak (VTi–VTe < 20%) and with leak (VTi–VTe ≥ 20%). Re-used devices showed a significantly lower leak rate than new devices (statistically significant; *p* = 0.003).

**Figure 5:**
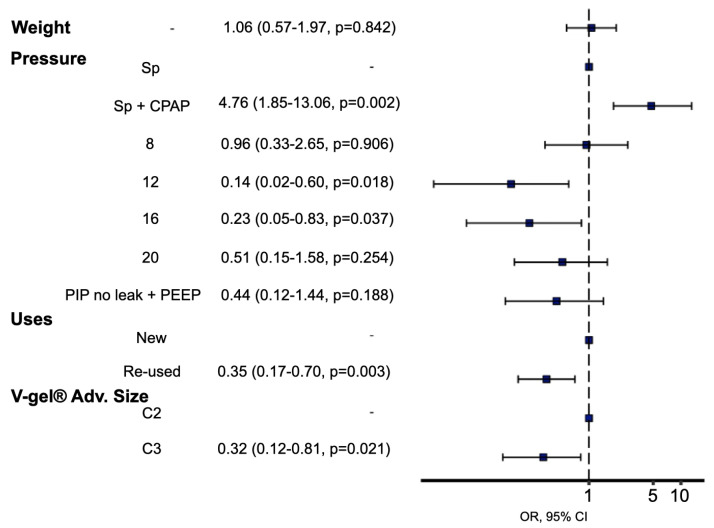
Relationships between leakage and number of V-gel^®^ advanced re-uses, different ventilatory settings, new/re-used devices and supraglottic device size. They were evaluated using a generalised linear model. Statistical significance was set at *p* < 0.05. Spontaneous + CPAP is significatively more likely to have leakage while using PIP of 12 cmH_2_O, 16 cmH_2_O and using C3 device size are significatively less likely to have leakage.

The proportion of cats exhibiting leakage under each ventilatory setting is shown in Table 2. The amount of leakage for each setting is shown in Figure 3. Re-used devices leaked significantly less than new devices (*p* = 0.003) (Figure 4). In the mixed-effects logistic regression, device re-use was associated with reduced leak probability (OR 0.35, 95% CI 0.17 to 0.70, *p* = 0.003). Relative to spontaneous breathing, CPAP increased leak probability (OR 4.76, 95% CI 1.85 to 13.06, *p* = 0.002), whereas PCV at PIP 12 and 16 cmH_2_O reduced it (OR 0.14, 95% CI 0.02 to 0.60, *p* = 0.018; and OR 0.21, 95% CI 0.05 to 0.85, *p* < 0.05, respectively). At PIP 20 cmH_2_O the reduction did not reach significance (OR 0.47, 95% CI 0.14 to 1.50, *p* > 0.05). No ventilatory setting × device condition interactions were significant. A random intercept for cat accounted for within-animal correlation. In addition, use of re-used devices, size C3 V-gel^®^ advanced was also associated with a lower likelihood of leakage (OR 0.32, 95% CI 0.12 to 0.81, *p* = 0.0021) (Figure 5).

**Figure 6:**
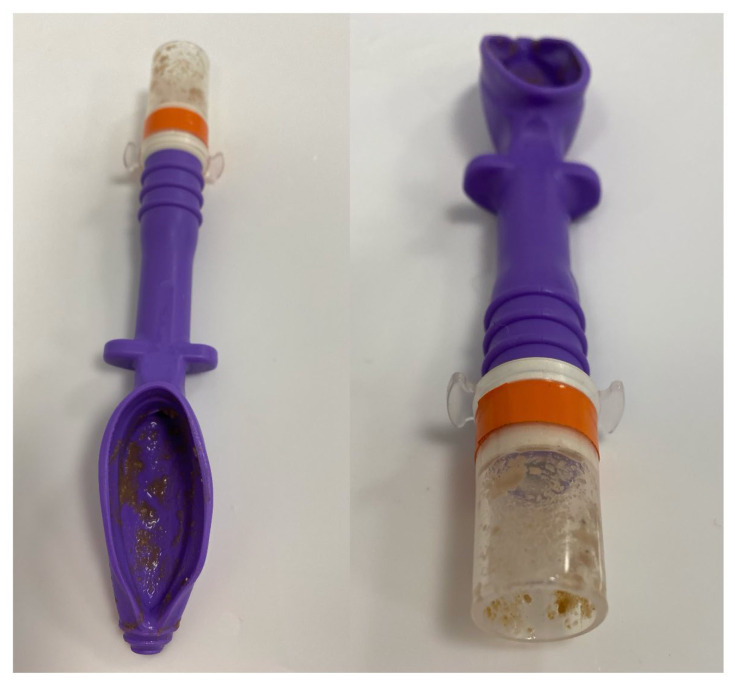
Picture showing one of the supraglottic device with stomach content inside the device.

Anaesthesia time was evaluated in 48 of the 52 animals. One cat was excluded due to severe regurgitation that led to euthanasia, another in which the supraglottic device was replaced by an endotracheal tube, and two animals that were found to be males (initially presumed to be females) and underwent castration. Regarding complications, 51 animals were included in the analysis, with only the cat intubated with an endotracheal tube being excluded. Device-related complications were recorded in a small number of cats. Pulmonary aspiration of gastric contents occurred in two cats; one was euthanized prior to recovery, and the other was discharged one week later after medical treatment. Mild regurgitant material within the device was observed in three cats (Figure 6). Ventilatory failure at the start of surgery occurred in one cat, prompting conversion to endotracheal intubation. No further complications were identified in relation to the use of the supraglottic device. Other complications unrelated to the use of the device included hypothermia, hypotension, and surgical complications such as uterine rupture and abdominal wall resuturing. The results for anaesthesia time and complications are summarised in Table 3.

## 4. Discussion

To the authors’ knowledge, this is the first clinical evaluation of the V-gel^®^ advanced in cats anaesthetised for abdominal surgery in which leaks were assessed across different ventilation modalities. During spontaneous ventilation, leakage occurred in 13% of cases. This contrasts with reports for the previous V-gel^®^ model, in which clinically relevant leakage during spontaneous breathing was not detected [15,16]. Given those earlier findings, the present result was unexpected and warrants confirmation in further clinical studies and, ideally, direct comparative trials between the two V-gel^®^ designs.

Overall, the clinical findings on leakage in the present study are broadly consistent with prior work using this device. Debuigne et al. [18] and Silvestre-Gómez et al. [19] assessed placement of the V-gel^®^ advanced by computed tomography (CT) in anaesthetised cats and reported that the seal was incomplete in a substantial proportion of cases, with the most frequent issue being inadequate closure at the oesophagus (65% and 72.5%, respectively). This contrasts with our dynamic measurements: although leakage was also detected by spirometry, the proportion was lower (13%). Debuigne et al. [17] further examined ventilation at a peak inspiratory pressure (PIP) of 8 cmH_2_O and observed increased gas within the oesophagus and stomach, inferring leakage into these compartments. In our study, only 12.7% of our animals showed leakage using the same PIP (8 cmH_2_O). Divergences between studies may reflect methodological differences (static CT imaging versus dynamic spirometric monitoring with different detection thresholds) and animal positioning; in our protocol all cats were in dorsal recumbency, and a breathing system holder was used to minimise device displacement, potentially improving pharyngeal apposition and reducing leakage. Nevertheless, this remains a hypothesis and should be tested in dedicated studies that concurrently compare imaging and ventilatory metrics across positions and ventilation modes.

Niyatiwatchanchai et al. [20] compared leakage in cats using the earlier V-gel^®^ under two modes of mechanical ventilation, volume-controlled ventilation (VCV) and pressure-controlled ventilation (PCV). They found no difference between modes in the proportion of animals exhibiting leakage, and the overall leakage rate 0.4%. The leakage rate found in cohort, considered just the CMV without PEEP, was 8.6%, still much higher than the previously reported data. These discrepancies may reflect superior placement by a consistent, experienced operator in that study (versus multiple inexperienced operators in ours) and/or design differences between device generations. These hypotheses warrant testing in a dedicated, adequately powered comparative study.

The proportion of cats with leakage increased to 41.8% when CPAP at 5 cmH_2_O was applied. In human anaesthesia, CPAP used with laryngeal masks increases mean airway pressure and lung volume [21], a change that could plausibly exacerbate leaks around a supraglottic seal. By contrast, leakage during controlled mechanical ventilation (CMV) was lower than reported in some veterinary studies [18], and, importantly, lower than during spontaneous ventilation in our cohort. This differs from findings with the earlier V-gel^®^ model [15]. One explanation could be that pressure-controlled ventilation improves apposition of the device, thereby enhancing the laryngeal seal and, potentially, the distal oesophageal seal. Adding 5 cmH_2_O of PEEP increased leakage relative to CMV without PEEP but remained lower than with spontaneous breathing; as other PEEP levels were not tested, no inference can be made about dose–response effects.

Notably, the lowest leak rates in our study were recorded at peak inspiratory pressures of 12 cmH_2_O (2.1%) and 16 cmH_2_O (6.5%), respectively. This contrasts with Szilágyi & Szilágyi [22], who reported substantial leakage at comparable PIP values (12–16 cmH_2_O) using V-gel^®^ advanced devices designed for dogs. Such divergent findings underscore the importance of avoiding cross-study comparisons between devices intended for different species, and, by extension, differing geometries and tissue interfaces, because design and species-specific fit can materially affect seal performance.

Mechanical ventilation in cats has been associated with increased anaesthesia-related mortality in a large, multicentre cohort study [23]. That analysis (14,962 cases across 198 centres) identified ventilation as an independent risk factor after adjustment for case mix, with ventilated cats exhibiting higher odds of death than non-ventilated cats. Potential mechanisms include the challenge of delivering accurate small tidal volumes, susceptibility to volutrauma/barotrauma at higher peak inspiratory pressures, and peri-ventilatory haemodynamic effects in this species. These considerations reinforce the need for cautious, lung-protective settings, meticulous monitoring, and judicious use of positive pressure in feline anaesthesia, and provide clinical context for the present findings across CPAP and pressure-controlled modes [23].

An unexpected observation was that re-used devices leaked significantly less than new devices. A plausible mechanism is material softening and improved conformity after initial use and subsequent cleaning/disinfection. Devices were re-used 2–7 times without obvious macroscopic deterioration, but the sample size was insufficient to determine whether the effect varies with cumulative re-use. Although the reprocessing protocol mirrored that used for endotracheal tubes in our institution, neither disinfection efficacy nor microstructural material changes were assessed. Some cleaning protocols commonly used for canine endotracheal tubes have been shown to be insufficient to ensure complete disinfection [24]. Accordingly, particular caution is warranted when selecting and validating the disinfection method. The device is marketed for single use in line with human hygiene standards, and our finding should not be interpreted as an endorsement of off-label re-use without appropriate validation and infection-control risk assessment.

With regard to placement, our results align with prior work demonstrating high insertion success and ease of use for V-gel^®^ devices [13,14]. Sedation with dexmedetomidine, methadone and ketamine provided adequate depth for placement in most cats; some required top-ups, which may reflect the challenging handling of colony animals and variable intramuscular administration by students. Placement of the device was readily achieved by students once an adequate plane of anaesthesia had been reached, in agreement with previous feline studies that also report the ease of use of this supraglottic airway device. The decision to place the SAD before intravenous catheterisation, and thus potentially before administering a hypnotic agent such as propofol or alfaxalone, was driven by both animal and operator factors. All cats were free-roaming colony animals that were difficult to handle prior to intramuscular sedation, and catheter placement was performed by students; securing the airway first was therefore preferred, with the option to deliver sevoflurane via the SAD if additional relaxation was required. We did not include a contemporaneous endotracheal intubation group, but previous studies reported lower induction requirements for V-gel^®^ advanced placement than for intubation [10,14,15]. Our clinical impression was that many V-gel^®^ advanced devices were placed successfully at anaesthetic depths that would likely have been insufficient for safe intubation.

The most significant complication observed was pulmonary aspiration in two cats. In one case, a fasting error led to aspiration of semi-solid material with radiographic right-middle-lobe consolidation and severe respiratory signs; euthanasia was elected prior to recovery due to poor prognosis. The other cat aspirated liquid gastric contents and was discharged after one week of supportive therapy. Leak testing was not performed in either case since these complications happened during surgery. Mild regurgitant material within the device, without any clinical signs, was noted upon removal in three cats without subsequent respiratory signs, consistent with previous reports [10,25]. Additionally, clinical gastric dilatation was observed at the end of surgery in ten animals, although this was not systematically recorded and could not be quantified. Similar observations have been reported [15] and raise the possibility that V-gel^®^ advanced may not achieve a complete oesophageal seal in all cats. High fresh gas flows could promote gastrointestinal insufflation and predispose to regurgitation, as suggested previously [12]. One episode of apparent upper-airway obstruction after device placement prompted conversion to endotracheal intubation; the obstruction persisted and was subsequently attributed to a breathing-system fault and thus was not considered device-related. No additional abnormalities were identified on capnography or pulse oximetry, and no further complications attributable to the supraglottic device were observed either intra-operatively or at removal. In human anaesthesia, supraglottic airway devices (particularly latest-generation models) have been used safely for abdominal surgery [5,6]. Airway protection is particularly important in situations with an increased risk of regurgitation, aspiration, or elevated intra-abdominal pressure. These factors become especially relevant in abdominal procedures, as patient positioning and surgical manipulation may promote reflux, and controlled mechanical ventilation at higher airway pressures, as in laparoscopic surgery, may be required. Based on our results, the V-gel^®^ advanced provided effective ventilation and oxygenation in anaesthetised cats, with no issues in 46 of 51 animals. In the remaining five cases, as previously mentioned, gastric material was observed within the device. Accordingly, while this device may represent a viable alternative to endotracheal intubation for these procedures, clinicians should recognise the potential risk of aspiration of oesophageal or gastric contents if an adequate laryngeal seal is not achieved. That said, use of an endotracheal tube does not itself eliminate aspiration risk or other complications [3,26,27,28]. Whichever device is chosen, optimal placement and maintenance should be ensured, and measures taken to minimise factors that predispose to regurgitation or other complications during anaesthesia.

This study has limitations. First, there were no other devices to compare (earlier V-gel^®^ model or endotracheal intubation), limiting between-device inferences. Second, the bodyweight range clustered such that only sizes C2 and C3 were evaluated; extrapolation to other sizes is not justified. Although size selection followed the manufacturer’s chart, overlap around the C2–C3 boundary means some cats may not have received the optimal size. Third, fresh gas flow rates were not standardised or captured; flow-dependent gastrointestinal insufflation could influence both leakage and regurgitation rates. Replication with controlled flow settings would be informative. Fourth, while anaesthetic machine, breathing system, monitoring, anaesthetic depth and animal position for testing were standardised, the final head/neck position was not recorded. In people, supraglottic device performance varies with head and body position under general anaesthesia during mechanical ventilation and with pressure support/PEEP [29,30]; a dedicated study would be required to quantify positional effects in cats. Finally, the number of cases included in this study was mainly determined by the number of animals available during the students’ clinical practice. As a result, some of the parameters evaluated may not have had sufficient statistical power to detect true differences. Nevertheless, the use of different statistical tests may have helped to mitigate this limitation.

## 5. Conclusions

V-gel^®^ advanced provided a practical and rapid means of securing the airway in cats undergoing abdominal surgery. Leakage was more common during spontaneous breathing and with CPAP than during CMV, and re-used devices exhibited lower leakage than new devices in this cohort. Complications included regurgitation and, rarely, aspiration, underscoring the importance of fasting verification, careful intra-operative monitoring, judicious fresh gas flows, and vigilance for gastrointestinal insufflation. Further controlled clinical studies, ideally including direct comparisons with the previous V-gel^®^ model and with endotracheal intubation, evaluation across device sizes, standardised gas flows, and assessment of head/neck position, are needed to define performance characteristics and clarify the potential for incomplete oesophageal sealing.

## Figures and Tables

**Table 1:** Body weight of the cats included in the study (mean ± SD), the percentage of cases requiring an additional anaesthetic dose to permit device placement, number of attempts required to place the supraglottic device (median [range]), number of times supraglottic devices were used (median [range]) and V-gel^®^ advanced size used are shown in the table.

Variable	Value
**Weight**	
Mean (SD)	2.88 (0.635)
**Top-up Dosing**	
No	31 (66%)
Yes	16 (34%)
**V-gel^®^ advanced Placement Attempts**	
Median [Min, Max]	1 [1, 2]
**V-gel^®^ advanced Re-uses**	
Median [Min, Max]	3 [0, 8]
**V-gel^®^ advanced Size**	
C2	34 (72.3%)
C3	13 (27.7%)

**Table 2:** Inspired volume (VTi), expired volume (VTe), absolute leak per breath (showed as median and minimum, maximum [Min, Max]), and percentage leakage for each ventilatory mode and setting studied (including the number of cases assessed for each setting). A loss of <20% between inspired and expired volumes was not considered a leak, whereas a difference ≥20% was classified as a leak. Spontaneous (Sp), Positive End Expiratory Pressure (PEEP), Peak Inspiratory Pressure (PIP), Continuous Positive Airway Pressure (CPAP), maximum pressure in which no leak was found (PIP no leak).

Ventilatory settings (Number of Cases)	Insp. Volume (VTi) ml Median [Min, Max]	Exp. Volume (VTe) ml Median [Min, Max]	leakage (%)
**Sp** **(46)**	11.3 [3.11, 19.1]	10.5 [3.11, 18.1]	<20%: 40 (87%) ≥20%: 6 (13%)
**Sp + 5 cmH_2_O CPAP** **(43)**	7.8 [2.67, 16.3]	6.56 [1.67, 15.1]	<20%: 25 (58.2%) ≥20%: 18 (41.8%)
**PIP 8 cmH_2_O** **(47)**	13.8 [6.88, 30]	12.8 [5.63, 29]	<20%: 41 (87.3%) ≥20%: 6 (12.7%)
**PIP 12 cmH_2_O** **(47)**	19.7 [10.3, 56]	19.3 [9.38, 54.5]	<20%: 46 (97.9%) ≥20%: 1 (2.1%)
**PIP 16 cmH_2_O** **(46)**	29 [13.8, 71.0]	27.5 [12.5, 68]	<20%: 43 (93.5%) ≥20%: 3 (6.5%)
**PIP 20 cmH_2_O** **(45)**	39.5 [16.6, 87.6]	38 [16.3, 85.4]	<20%: 39 (86.7%) ≥20%: 6 (13.3%)
**PIP no leak +** **5 cmH_2_O PEEP** **(42)**	25.3 [11, 61.1]	25.3 [9.33, 51.6]	<20%: 37 (88.1%) ≥20%: 5 (11.9%)
**Overall** **(316)**	18.6 [2.67, 87.6]	17.8 [1.67, 85.4]	<20%: 271 (85.8%) ≥20%: 45 (14.2%)

**Table 3:** Results are presented as anaesthesia time (minutes; median [minimum, maximum]), main complications associated with the use of the supraglottic device, and other complications not related to its use.

Variable	Value
**Anaesthesia time (min)**	85 [35, 160]
**Complications related to V-gel ^®^ advanced**	
Gastroesophageal content inside the device	9.8% (5/51)
Death (euthanasia)	1.9% (1/51)
Aspiration pneumonia	1.9% (1/51)
**Other complications no related to V-gel ^®^ advanced**	
Hypothermia	3.9% (2/51)
Hypotension	5.9% (3/51)
Surgical complications	3.9% (2/51)

## Data Availability

The raw data supporting the conclusions of this article will be made available by the authors on request.
